# A glimpse at short-term controls of evapotranspiration along the southern slopes of Kilimanjaro

**DOI:** 10.1007/s10661-017-6179-9

**Published:** 2017-08-23

**Authors:** Florian Detsch, Insa Otte, Tim Appelhans, Thomas Nauss

**Affiliations:** Environmental Informatics, Faculty of Geography, Philipps-Universität Marburg, Deutschhausstr. 12, 35032 Marburg, Germany

**Keywords:** Evapotranspiration, Land-use change, Global climate change, Elevation gradient, Surface-layer scintillometer, Kilimanjaro

## Abstract

Future climate characteristics of the southern Kilimanjaro region, Tanzania, are mainly determined by local land-use and global climate change. Reinforcing increasing dryness throughout the twentieth century, ongoing land transformation processes emphasize the need for a proper understanding of the regional-scale water budget and possible implications on related ecosystem functioning and services. Here, we present an analysis of scintillometer-based evapotranspiration (ET) covering seven distinct habitat types across a massive climate gradient from the colline savanna woodlands to the upper-mountain *Helichrysum* zone (940 to 3960 m.a.s.l.). Random forest-based mean variable importance indicates an outstanding significance of net radiation (*R*_net_) on the observed ET across all elevation levels. Accordingly, topography and frequent cloud/fog events have a dampening effect at high elevations, whereas no such constraints affect the energy and moisture-rich submontane coffee/grassland level. By contrast, long-term moisture availability is likely to impose restrictions upon evapotranspirative net water loss in savanna, which particularly applies to the pronounced dry season. At plot scale, ET can thereby be approximated reasonably using *R*_net_, soil heat flux, and to a lesser degree, vapor pressure deficit and rainfall as predictor variables (*R* ^2^ 0.59 to 1.00). While multivariate regression based on pooled meteorological data from all plots proves itself useful for predicting hourly ET rates across a broader range of ecosystems (*R* ^2^ = 0.71), additional gains in explained variance can be achieved when vegetation characteristics as seen from the NDVI are considered (*R* ^2^ = 0.87). To sum up, our results indicate that valuable insights into land cover-specific ET dynamics, including underlying drivers, may be derived even from explicitly short-term measurements in an ecologically highly diverse landscape.

## Introduction

Land-use change influences the local to regional-scale water balance (Jung et al. 2010), including precipitation on the credit side and surface run-off, ground-water flow, evaporation, and transpiration on the debit side (DeFries and Eshleman 2004; Foley et al. 2005). Deforestation concomitant with a declining leaf area index, for instance, results in decreased evapotranspiration (ET) in favor of higher surface temperatures and a higher temporal variability of related energy fluxes (Biudes et al. 2015). Global environmental change, on the other hand, is likely to have a crucial impact on the trans-regional water budget (Glenn et al. 2010) which is particularly applicable to tropical mountain regions and their unique ecosystems (Buytaert et al. 2011).

In this scope, the species-rich Kilimanjaro region is one of the most famous hot spots of global warming in the tropics (Thompson et al. 2002). Of the numerous climate zones, and hence vegetation zones, aligned along the mountainsides (Duane et al. 2008), changing climate conditions especially affect the upper-mountain regions starting from 3,000 meters above sea level (m.a.s.l.) upwards (Torbick et al. 2009). Moreover, the associated increase in evaporative demand over East Africa (Cook et al. 2014) presumably amplifies net water loss throughout the drought-prone area (Afifi et al. 2014).

Simultaneously, extensive land-use change in the densely vegetated foothills accounted for an expansion of cultivated land from 54% in 1973 to 63% in 2000 (Misana et al. 2012) at the expense of natural vegetation (Hemp 2006a)—a trend that seemingly endured beyond the turn of the millennium (Tracewski et al. 2016). While human intervention primarily affects ecosystems outside the protective realms of Kilimanjaro National Park, natural disturbance regimes profoundly modify the upper-mountain vegetation structures. Hemp (2005), for instance, demonstrated the fire-driven expansion of *Erica* bush at the expense of domiciled *Erica* forest.

From a hydrological viewpoint, such massive conversion processes in an area facing a steadily increasing population pressure (Misana et al. 2012) severely affect the regional water cycle, perhaps even more so than climate change (Hardwick et al. 2015). Although an appropriate quantification of land cover-specific water release through ET is of vital importance (Savage 2009), only little data on biosphere-atmosphere water exchange has been available until now. In this regard, the surface-layer scintillometer (SLS) method (Odhiambo and Savage 2009) might help to overcome limitations associated with the highly heterogeneous terrain as it only requires several tens of meters to operate properly. Weiss (2002), for instance, successfully applied the SLS method to derive turbulent fluxes of sensible heat and momentum over complex terrain in an alpine valley. In the African context, Savage (2009) and Odhiambo and Ain (2011) performed scintillometer-based measurements of evaporation over an open grassland site and demonstrated broad agreement with results obtained from eddy covariance (EC).

Encouraged by such flagship studies, we would like to take a step towards assessing the implications of ET and its underlying driving forces on ecosystem functioning and services in the highly fragile Kilimanjaro region. Therefore, the aims of our study are formulated as follows:

- Firstly, we run short-term SLS-based ET measurements over numerous natural and disturbed habitat types characteristic for the Kilimanjaro region, thus establishing an ET-elevation gradient spanning a height range of more than 3000 m.
- Secondly, we analyze the relative importance of short-term meteorological drivers on ET (e.g., temperature, radiation, rainfall) and establish a link between ET-elevation patterns and long-term eco-climatological influences.
- Thirdly, we assess the degree to which vegetation characteristics per ecosystem are capable of contributing to the complex interplay between meteorological factors and the observed net water loss through ET.

## Material and methods

### Study area and sampling design

**Fig. 1:**
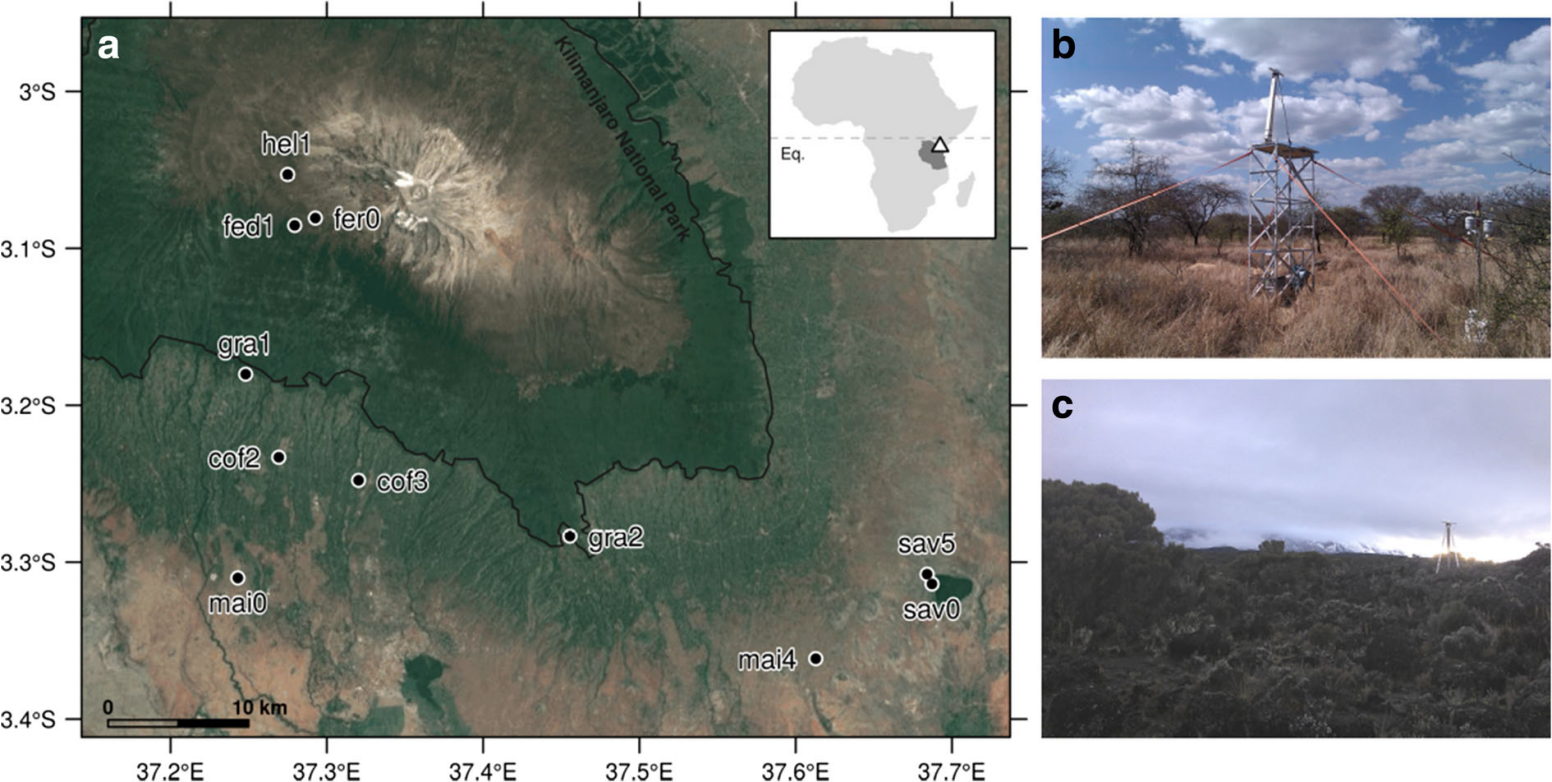
**a** Location of Kilimanjaro (top-right panel) and sampling plots superimposed upon a satellite image of the study area (EPSG:4326; Google and TerraMetrics (2017)). **b**, **c** Impressions from the field campaign at dry-season sav5 and hel1, respectively

The Kilimanjaro region is located in the north-east of Tanzania and spans an elevation gradient from the colline savanna plains (∼ 700 m.a.s.l.) to the glaciated areas encircling Kibo summit (5895 m.a.s.l.). Its equatorial daytime climate is shaped by the passing of the intertropical convergence zone, with more than half of the annual rainfall occurring during the so-called long-rains (March to May; Appelhans et al. (2016)) as a consequence of moist south-easterly winds (Oettli and Camberlin 2005). While annual precipitation amounts to more than 2500 mm in the southern montane forest belt, the northern mountainside receives hardly more than 1000 mm (Hemp 2006c). The mountain’s belt-like vegetation zonation (Fig. 1a) is characterized by major land-cover transitions at short horizontal distances resulting from changing climate conditions and anthropogenic interference (Buytaert et al. 2011).

Embedded in a German-Tanzanian research program, a total of 65 sampling sites have been selected with respect to plant taxonomic aspects along the southern slopes (Peters et al. 2016, and Supplementary Fig. 1 therein). The resulting altitude gradient spans more than 3600 m and includes a variety of natural and disturbed habitat types stretching from the intensely cultivated savanna woodlands to the lower alpine *Helichrysum* belt (Röder et al. 2016). For detailed information about the sampling design, meteorological, and vegetation characteristics of the single habitats, the reader is kindly referred to the profound works by Hemp (2006c), Appelhans et al. (2016), Röder et al. (2016), and Peters et al. (2016).

In order to investigate habitat-specific ET and its underlying drivers within the scope of this research project, our study features a subset of the land-cover types included therein, namely

- at the colline level (700–1000 m.a.s.l.), (i) savanna woodland (“sav”) characterized by *Acacia-Commiphora* vegetation with average canopy heights of 4.6 m (Fig. 1b; Ensslin et al. (2015)), sampled both during the dry and rain season, and (ii) green maize fields (“mai”; *Zea mays*) close to tasseling with heights up to 1.8 m;
- at the submontane level (1000–1800 m.a.s.l.), (i) coffee plantations (“cof”) with pruned (0.3–0.5 m) and mature coffee shrubs (1.5–2.5 m) at cof3 and cof2, respectively, shaded by large *Albizia* trees (e.g., *A. schimperiana*; Hemp (2006b)), and (ii) frequently cut grasslands (“gra”) with explicitly low canopies (≤ 0.2 m);
- at the subalpine level (3100–4000 m.a.s.l.), (i) natural *Erica* forest (“fer”) dominated by *E. excelsa* and up to 10 m high, and (ii) primarily fire-disturbed *Erica* bushland (“fed”) dominated by *E. arborea* and *E. trimera* (≈ 1.5 m; Hemp (2005));
- at the lower alpine level (4000–4500 m.a.s.l.), low-canopy *Helichrysum* cushion (“hel”; Fig. 1c) with *H. newii* and *H. citrispinum* as “climatic climax vegetation” (Hemp 2009, p. 1017).

Note that due to high canopies (Odhiambo and Savage 2009), neither the famous Chagga homegardens at the submontane level (plots located from 1170 to 1832 m.a.s.l.; Hemp (2006b)) nor the montane forest belt (plots located from 1737 to 3015 m.a.s.l.; Appelhans et al. (2016)) could be considered within the scope of this study. Moreover, ease of accessibility and topographic suitability limited measurement capabilities in high elevations. Nonetheless, appropriate plots for all designated habitat types could be established (Fig. 1a), of which some topographic and beam path characteristics are given in Table 1. Beam path lengths ranged between 64 to 120 m and were primarily determined by (i) habitat size at low elevations, class “sav” excluded and (ii) topographic suitability at high elevations. Path heights, on the other hand, were adapted to canopy height and ranged from 2 to 5.4 m above ground.

**Table 1:** Date range, topography and beam path characteristics per plot. ‘(d)’ indicates dry-season measurements

PlotID	Date range	Topography			Beam path		
	Start (no. of days)	Elevation	Slope angle	Aspect	Length	Height	Inclination
		(m.a.s.l.)	(^∘^)	(^∘^)	(m)	(m)	(^∘^)
fer0	2014329 (6)	3956	17.2	196	100	4	− 10.7
hel1	2014335 (5)	3849	9.7	310	120	3	0.8
fed1	2014323 (6)	3498	17.2	264	70	4	− 7.8
gra2	2014076 (4)	1754	20.4	94	70	2	− 0.5
gra1	2014056 (4)	1742	6.8	146	64	2	4.1
cof2	2014081 (5)	1353	2.8	175	68	5.4	− 0.3
cof3	2014070 (3)	1288	18.3	270	103	2.2	4.7
mai0	2014137 (5)	1010	1	177	71	5.3	0.5
mai4	2014133 (5)	962	1.5	273	79	5.3	− 1.6
sav0 (d)	2014259 (5)	953	0.8	23	104	5.4	−0.8
sav0	2014125 (5)	953	0.8	23	77	5.4	− 0.8
sav5 (d)	2014255 (5)	943	1.2	13	94	5.4	− 0.9
sav5	2014129 (5)	943	1.1	13	79	5.3	− 0.9

### Theoretical and technical background

As regards ground-based remote sensing systems as an alternative to conventional methods such as EC, scintillometer-based techniques (Kite and Droogers 2000; Meijninger and de Bruin 2000) have lately received considerable attention regarding their capabilities of yielding reliable ET estimates in rather short time intervals. In this context, the comprehensive works by Odhiambo and Savage (2009) and Savage (2009) provide essential information on theoretical background, history, and relevant applications of the SLS method. Briefly, the general principle of scintillometry assumes that turbulent fluctuations of air temperature (*T*_a_), and to a lesser degree, relative air humidity (rH) induce small-scale fluctuations of the refractive index of air. This, in turn, affects a laser beam propagating along a given path and hence allows the derivation of turbulence-related parameters from the resulting intensity fluctuations.

The continuous monitoring of the radiation signal requires a stationary field setup with a transmitter and receiver unit typically positioned 50 to 300 m apart from each other. Among the advantages associated with the SLS technique (Odhiambo and Savage 2009), Nakaya et al. (2007) highlight the representative nature of the path-averaged flux measurements due to the larger source area, which makes the technique particularly suitable for small research sites. While requiring no simultaneous wind speed measurements in order to derive ET, the SLS results show a more plausible behavior on short time scales as compared to EC while maintaining consistent data quality (Thiermann and Grassl 1992).

The herein presented data is derived from a dual-beam SLS of the type SLS40 (Scintec AG, Rottenburg, Germany). Complementing factory-certified calibration parameters for wavelength and separation of the laser beam (Van Kesteren et al. 2014), additional calibration is carried out in the field through quantifying the background signal and crosstalk coefficients before launching the actual measurement. An accompanying automated weather station (AWS; Scintec (2013a)) records *T*_a_ and rH, from which the vapor pressure deficit (VPD) as an important explanatory variable of ET in mountainous terrain (Nullet and Juvik 1994) can be calculated, at 1-min intervals. Further parameters collected by the AWS are air pressure (*p*), net radiation (*R*_net_), soil heat flux (*S*), and rainfall, among others (Scintec 2013c). Note that wind speed and direction were not collected within this scope.

### Estimation of latent heat flux

Odhiambo and Savage (2009) argue that canopy-related flux terms are negligible for short and open canopies and, moreover, advection can be left unattended when dealing with rather homogeneous surfaces. Considering the characteristics of the selected research plots (“Study area and sampling design”), the application of the shortened energy balance equation to derive the latent heat flux (LE; as a surrogate for ET) from

1 $$ R_{\text{net}} = \textit{LE} + H + S  $$

therefore seems justified (Odhiambo and Savage 2009). Here, *R*_net_ is net radiation, whereas *H* and *S* are sensible and soil heat flux, respectively. It becomes evident that the SLS-based derivation of *H* allows the estimation of LE given that *R*_net_ and *S* are available. Note, however, that Eq. 1 cannot be closed entirely which, in a more practical sense, means that the residual value (i.e., *R*_net_ – *H* – *S*) and the actual LE are not exactly the same (Tagesson et al. 2015). Despite this well-known closure issue in boundary-layer meteorology (e.g., Foken (2008)), Tagesson et al. (2015) underline that averaged daily flux estimates calculated from Eq. 1 may still provide valuable information within the scope of eco-climatological research (e.g., model validation).

### Data processing

The SRun software for automated SLS data retrieval combines simultaneous optical and meteorological recordings at 1-min intervals (Scintec 2013b), from which hourly ET rates (mm/h) are calculated. Measurement gaps introduced during times of power shortage, strong wind and fog add up to 7.2% of all hourly values and are partly refilled using a random forest algorithm (RF; Breiman (2001)). While filling missing data introduced by sensor instabilities based on concomitantly recorded environmental variables is generally considered unproblematic (Goulden et al. 2012), gaps resulting from fog involve a number of unknowns (Dawson 1998) and are hence not recomputed.

Internal validation of the implemented gap-filling routine consists of tenfold cross validation (CV) performed upon randomly selected training data (75% of all complete records). For a varying number of split variables, the model with the smallest root mean square error (RMSE_CV_) is used to predict the remaining 25% of the complete records. The thus derived ET rates are subsequently compared with the test data by calculating the test RMSE (RMSE_T_), mean absolute error (MAE_T_) and *R* T2. The entire procedure is carried out ten times for each plot separately to determine the optimum number of split variables and the mean values of all error metrics.

Complementing model validation, the mean variable importance of each meteorological input in terms of predicting ET is determined. Briefly, RF-based variable importance can be derived through randomly permuting the values of a given predictor while leaving all remaining variables unchanged (Altmann et al. 2010). The resulting changes in prediction error (as seen from MAE in regression-based scenarios) serve as a variable-specific measure for the mean decrease in model accuracy (Liaw and Wiener 2002), and therefore, deploying such an approach is expected to provide deeper insights for our analysis.

### Satellite data

The amount to which vegetation characteristics of ecosystems contribute to ET-elevation relationships has been broadly discussed in the literature. In order to assess potential impacts across multiple land covers in the Kilimanjaro region, our analysis is complemented by satellite-borne estimates of the Normalized Difference Vegetation Index (NDVI) derived from the Moderate Resolution Imaging Spectroradiometer (MODIS) aboard the Terra and Aqua satellites. Being a measure of photosynthetic activity at the Earth’s surface, the index distinctly varies between ecosystems and closely correlates with daytime land surface temperature (LST; Maeda and Hurskainen (2014)) and ET (Maeda et al. 2011). It is calculated from (Tucker 1979)

2 $$ \text{NDVI} = (\rho_{\text{NIR}} - \rho_{\text{red}}) / (\rho_{\text{NIR}} + \rho_{\text{red}})  $$

where *ρ*_NIR_ and *ρ*_red_ are reflectances from the near-infrared (841–876 nm) and red MODIS bands (620–670 nm), respectively.

Terra and Aqua-MODIS provide best value composites of two selected “Vegetation Indices” (VI), namely Enhanced Vegetation Index (EVI) and NDVI, at a spatial and temporal resolution of 250 m and 16 days, respectively (MOD/MYD13Q1 V006), released with a time lag of 8 days and downloaded from the Land Processes Distributed Active Archive Center (LP DAAC; https://lpdaac.usgs.gov/). Following two-fold quality control based on the companion “pixel reliability” and “VI quality” layers to retain reliable pixels only, the two datasets are merged into a continuous 8-day time series. Finally, a modified Whittaker Smoother (Atzberger and Eilers 2011) is applied for down-weighting remainders of negatively biased, low-confidence values in favor of a non-disturbed vegetation signal (Detsch et al. 2016).

## Results

### Evaluation of ET gap filling

The calculated training and test statistics (Table 2) confirm that the RF-based algorithm used to refill missing hourly ET rates based on simultaneous meteorological recordings performs reasonably well. As for model training, RMSE_CV_ ranges from 0.03 to 0.07 mm/h, whereby the associated values of *R* CV2 (0.58 to 0.99) indicate considerably strong linear relationships. A similar picture is drawn by the test statistics, where RMSE_T_ and MAE_T_ also range from 0.03 to 0.07 and 0.01 to 0.04 mm/h, respectively, and *R* T2 lies between 0.64 to 0.99.

**Table 2:** Plot-specific training and test statistics of the random forest-based gap filling

Plot ID	Training		Testing		
	RMSE_CV_	*R* CV2	MAE_T_	RMSE_T_	*R* T2
fer0	0.03 ± 0.006	0.96	0.01 ± 0.001	0.03 ± 0	0.96
hel1	0.03 ± 0.005	0.97	0.01 ± 0.001	0.03 ± 0	0.97
fed1	0.07 ± 0.007	0.85	0.03 ± 0.002	0.07 ± 0.001	0.85
gra2	0.03 ± 0.002	0.99	0.01 ± 0.001	0.03 ± 0	0.99
gra1	0.06 ± 0.011	0.93	0.02 ± 0.002	0.05 ± 0.001	0.94
cof2	0.07 ± 0.009	0.94	0.04 ± 0.002	0.06 ± 0.001	0.94
cof3	0.03 ± 0.003	0.99	0.02 ± 0.001	0.03 ± 0	0.99
mai0	0.05 ± 0.005	0.96	0.02 ± 0.001	0.04 ± 0	0.96
mai4	0.04 ± 0.004	0.97	0.02 ± 0.001	0.04 ± 0	0.97
sav0 (d)	0.05 ± 0.003	0.76	0.02 ± 0.001	0.05 ± 0	0.77
sav0	0.07 ± 0.011	0.9	0.03 ± 0.002	0.07 ± 0.001	0.9
sav5 (d)	0.05 ± 0.014	0.58	0.02 ± 0.002	0.06 ± 0.001	0.64
sav5	0.04 ± 0.004	0.85	0.03 ± 0.001	0.04 ± 0	0.86

Included are the root mean square error (RMSE), coefficient of determination (*R* ^2^) and, in the case of model testing, mean absolute error (MAE). Subscripts “CV” and “T” signify results from model training and testing, respectively, whereas “(d)” indicates dry-season measurements

**Fig. 2:**
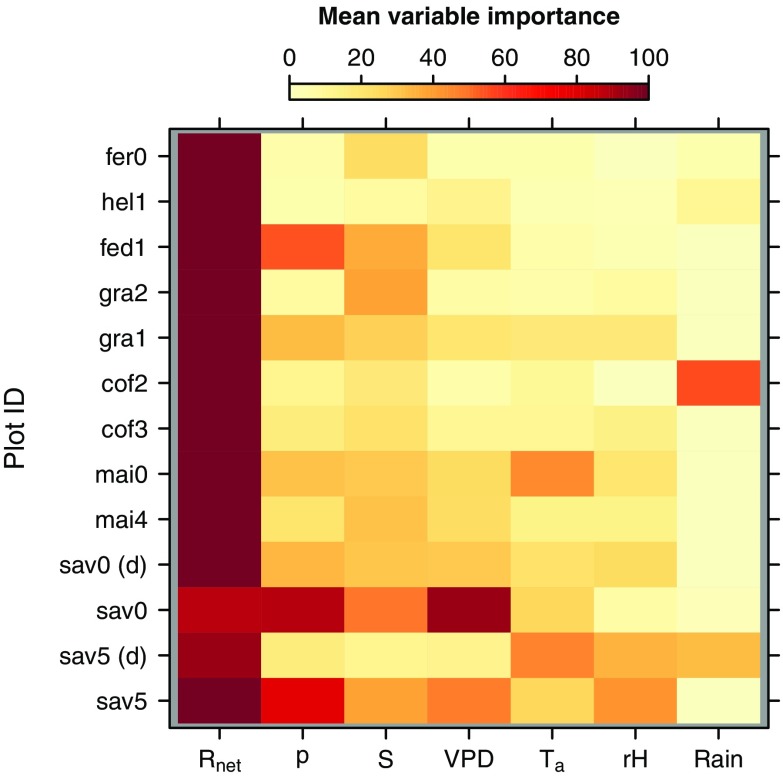
Mean relative variable importance per sampling plot (sorted from left to right in descending order of overall importance). *R*_net_ net radiation, *p* air pressure, *S* soil heat flux, VPD vapor pressure deficit, *T*_a_ air temperature, rH relative humidity. “(d)” indicates dry-season measurements

Complementing the training and test statistics, the corresponding mean variable importance from internal CV (Fig. 2) indicates that *R*_net_ is the most important parameter for explaining variations in ET. While *p*, *S*, and VPD follow in descending order, *T*_a_ and rH—from which VPD is calculated—play only a minor role. Finally, the relative importance of rainfall is virtually zero across all plots, with two minor exceptions (sav5 (d), cof2).

### Elevation profiles

**Fig. 3:**
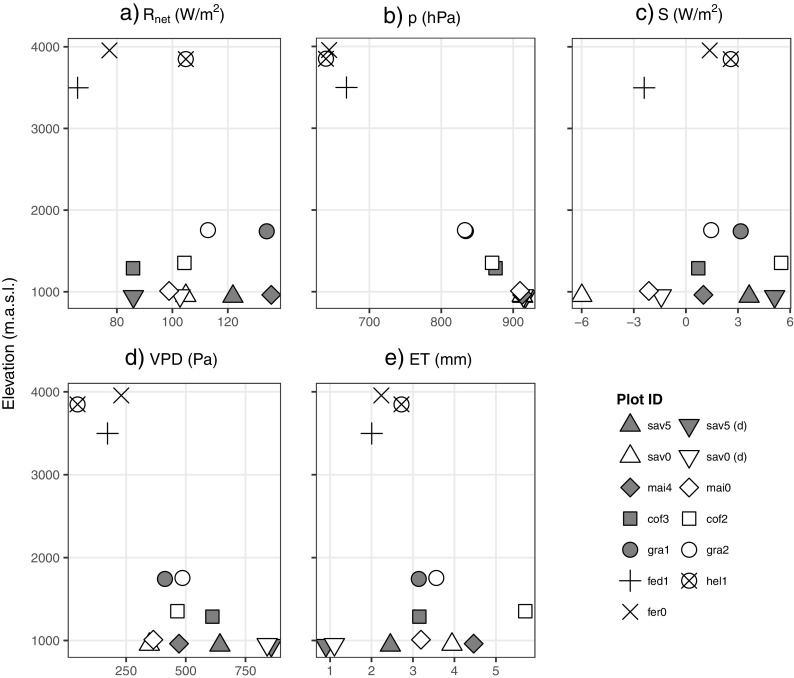
Elevation profiles of mean daily **a** net radiation (*R*_net_), **b** air pressure (*p*), **c** soil heat flux (*S*), **d** vapor pressure deficit (VPD), and **e** evapotranspiration (ET). In the legend, “(d)” indicates dry-season measurements, and point shapes and fill colors signify different land covers and plots per land cover, respectively

In order to assess the actual influence of the single meteorological drivers, further analyses on their short-term interplay with ET are required. As a starting point, Fig. 3 depicts the elevation profiles of plot-specific mean daily values of the four most relevant driving factors.

*R*_net_ lacks a clear altitude gradient as it varies considerably between plots even at similar elevation levels (Fig. 3a). It peaks at the colline maize (mai4, 135.7 W/m^2^) and submontane grassland level (gra1, 134.0 W/m^2^), whereas smaller values become evident from both low (e.g. sav5 (d), 86.0 W/m^2^) and high elevations (e.g. fed1, 65.8 W/m^2^). Remarkably, dry- and wet-season measurements at sav5 clearly differ, whereas no such seasonal pattern is evident from sav0. On the other hand, *p* reveals a much more uniform distribution as it linearly declines with elevation from savanna (sav5, 916.9 hPa) to *Helichrysum* (hel1, 639.6 hPa; Fig. 3b). Rather similar to *R*_net_, a considerably diverse picture is drawn by *S*, which cannot easily be subdivided into elevation levels and hence requires further analysis (Fig. 3c). Interestingly, two out of three high-elevation plots (hel1, fer0) show positive flux rates, whereas negative fluxes become evident from fed1. At the low-lying sites, the coffee and grassland habitats as well as sav5 (wet and dry) reveal positive flux rates, whereas sav0 (wet and dry) and maize are characterized by negative or only minor positive fluxes. Finally, the VPD-elevation relationship closely tallies with a strong linear decrease of *T*_a_ with elevation (Appelhans et al. 2016, and Fig. 6 therein) and reveals highest and lowest deficits at dry-season sav5 (858.1 Pa) and hel1 (46.6 Pa), respectively (Fig. 3d).

Depicted in Fig. 3e is the determined ET-elevation gradient which vaguely resembles a combination of the described progression curves of *R*_net_ and *S*. Accordingly, the highest ET rates are observable at cof2 (5.7 mm/day) that decline both uphill (fed1, 2.0 mm/day) and downhill (sav5, 2.5 mm/day). By far, the smallest rates become evident during the dry season in savanna (up to 0.9 mm/day at sav5).

For all variables displayed in Fig. 3a–d, including rainfall, univariate linear models are fitted to estimate the degree of variation explained in the observed ET (Table 3). Clearly, *R*_net_ is capable of explaining the broadest range of variance (*R* ^2^ 0.26 to 0.99), the sole exceptions being dry-season sav5 as well as cof2. *S* follows closely behind and reveals very similar correlations as *R*_net_ (*R* ^2^ 0.22 to 0.85), again with the aforementioned exceptions. VPD proves to be an important co-explanatory variable at least at some of the colline and submontane plots (up to *R* ^2^ = 0.57 at mai0) without following a particular habitat-related pattern. At higher elevations, by contrast, the vapor pressure gradient seems to be of no further relevance. Despite designated runner up in terms of RF-based variable importance, *p* generally has only little explanatory power for ET across all elevation levels. Rainfall plays a particular role at dry-season sav5—where the *R*_net_-based *R* ^2^ drops to zero—and, to a lesser extent, at cof2 and sav0, while no or only minor showers occurred at the remaining plots.

**Table 3:** Plot-specific coefficients of determination (*R* ^2^) derived from univariate and multivariate linear regression against hourly ET rates. Abbreviations are the same as in Figs. 2 and 3

PlotID	*R* ^2^(univariate)					*R* ^2^ (multivariate)
	*R*_net_	*p*	*S*	VPD	Rain	
fer0	0.70	0.09	0.44	0.06	0.10	0.85
hel1	0.88	0.02	0.49	0.03	0.02	0.96
fed1	0.56	0.14	0.26	0.02	0.22	0.89
gra2	0.99	0.03	0.85	0.27	0	1
gra1	0.97	0.11	0.82	0.46	0	0.99
cof2	0.11	0.17	0.10	0.02	0.32	0.63
cof3	0.96	0	0.70	0.16	0	0.98
mai0	0.84	0.03	0.67	0.57	0.03	0.89
mai4	0.75	0	0.68	0.24	0.07	0.87
sav0 (d)	0.83	0.03	0.56	0.14	0	0.88
sav0	0.26	0.02	0.22	0.04	0.29	0.62
sav5 (d)	0.01	0	0	0.04	0.47	0.59
sav5	0.80	0	0.71	0.52	0	0.81

The results obtained from multivariate linear regression (Table 3, right column) confirm that, when taking all of the aforementioned variables into account, ET may be reasonably estimated at plot scale in most cases (up to *R* ^2^ = 1 at gra2). Moreover, when pooling all data available and building one multivariate model for all plots, a comfortably high value of *R* ^2^ = 0.71 remains. Still, some deviations downwards persist at plot scale, which particularly include dry-season sav5 as well as cof2 and sav0, thus pointing towards additional influence factors that have not been considered so far.

### Vegetation characteristics

**Fig. 4:**
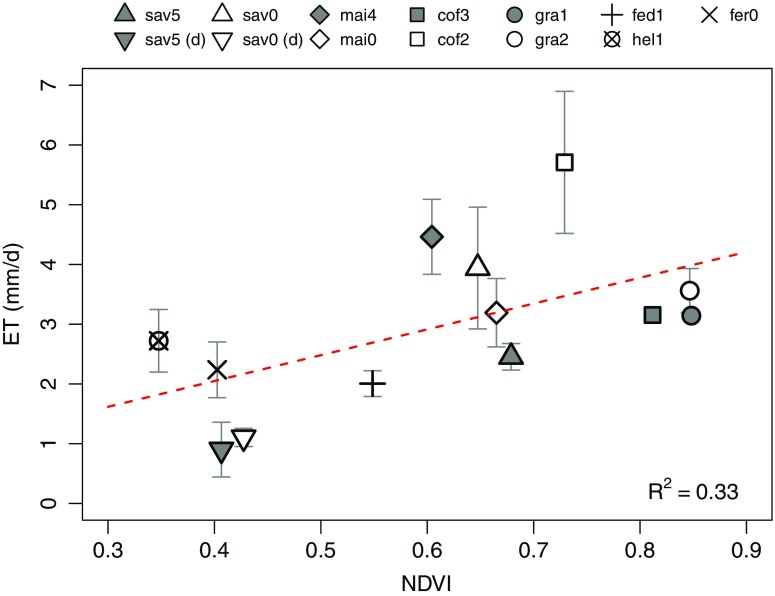
Relationship between average daily ET and NDVI on a plot basis. In the legend, “(d)” indicates dry-season measurements, whereas point shapes and fill colors identify different land covers and plots per land cover, respectively. Included are plot-specific standard errors (gray solid) and the overall linear function (red dashed)

In terms of vegetation characteristics, the highest NDVI values occur at the grassland sites (≈ 0.85; Fig. 4) and at cof3 (0.81), which moderately decrease towards cof2, the two maize fields and the rain-season savanna measurements. With the exception of fed1, the upper-mountain habitats and the dry-season savanna measurements reveal explicitly smaller values between 0.35 and 0.43. Despite the short measurement periods, the corresponding ET-NDVI relationship proves itself noticeably linear (*R* ^2^ = 0.33). Plot-specific standard errors thereby tend to increase towards the points lying above the fitted curve, suggesting considerable fluctuations of daily ET rates at otherwise constant NDVI values.

In order to establish a link between vegetation and meteorological characteristics, we incorporate the determined linear ET-NDVI relationship into the initially meteorologically driven multivariate model to estimate ET from data pooled across all plots (“Elevation profiles”). This time, however, hourly meteorological recordings are combined into average daily values which should improve comparability with our 8-day MODIS NDVI dataset. While the corresponding *R* ^2^ of the solely meteorology-driven model thereby increases to 0.78 owing to this temporal aggregation alone (as compared to *R* ^2^ = 0.71 for hourly observations), including the NDVI leads to considerable additional gains of *R* ^2^ = + 0.09 (or *R* ^2^ = 0.87).

## Discussion

### Meteorological controls on ET

#### Short-term controls

Clearly, *R*_net_ is the superior short-term meteorological driver of ET at plot scale (Fig. 3). This becomes particularly obvious from high-elevation habitats, where higher cloud/fog frequencies (Buytaert et al. 2011) restrain the amount of available energy and hence attenuate ET. At the low-lying plots, by contrast, energy is presumably not a limiting factor. The outstanding role of *R*_net_ is further evidenced from plot-based linear regression (Table 3), where it explains the largest amount of ET variance without taking other variables into account. Similar ET-elevation gradients have been reported previously, e.g., by Goulden et al. (2012) for California’s Sierra Nevada, and in this context, the amount of available energy has been identified as a major driver of ET (Nullet and Juvik 1994). In addition, Camberlin et al. (2014) reported an outstanding impact of global radiation not only on actual but also on potential ET (ET_0_) along elevation gradients at Mt. Kenya. Moving along horizontal rather than vertical climate gradients, Biudes et al. (2015) and Negrón Juárez et al. (2007) found an equally strong dependency of ET on radiative input during the wet and dry season, respectively, in Brazil. Considering this flood of studies coming to roughly the same conclusion, we see the validity of our short-term approach, and hence, our interpretation of *R*_net_ being the primary driver of ET in the study area confirmed.

In a slightly attenuated form, these findings also apply to *S* which is directly linked to *R*_net_ and LE through the surface energy balance (Eq. 1). Possibly owing to their joint key role in determining ET (Allen et al. 1998), our measurements indicate a strong statistical relationship between *S* and *R*_net_ across all sampling sites (*R* ^2^ from 0.82 to 0.97; data not shown). While this is well in agreement with findings from West Africa (Kakane 2004), the fact that *S* generally makes up only a smaller fraction of *R*_net_ (e.g., Da Rocha et al. (2004)) is likely responsible for the slightly smaller amounts of explained variance in observed ET (Table 3). Although the underlying *S* to *R*_net_ ratio considerably varies with land-cover type and season, which has also been one of the key outcomes in Kakane (2004, and references therein), our results obtained from univariate regression hence lead us to the conclusion that, complementing *R*_net_, *S* is an important co-explanatory variable for plot-based ET in the region.

In fact, it is only at those plots where precipitation occurred during our measurements that the relative importance of *R*_net_ and *S* declines (Table 3). In particular, this applies to dry-season sav5 where the impact of *R*_net_ and *S* practically approaches zero, whereas ET is governed almost exclusively by sudden rainfall events. Similar findings were, e.g., reported from semi-arid grassland and shrubland sites (Nagler et al. 2007), where peak ET rates occurred simultaneous to precipitation events and, moreover, long-term ET could be best approximated using rainfall in combination with EVI (*R* ^2^ = 0.74). *R*_net_ and *T*_a_, on the other hand, were only of subordinate importance.

As indicated by previous studies on evaporation profiles of tropical mountains (e.g., Nullet and Juvik (1994)), linear modeling also suggests that VPD might be an essential co-factor impacting ET at least at some of our plots. However, greater uncertainties remain as regards a proper interpretation of this interesting finding as higher explanatory power is restricted to some of the lower plots only. At the same time, the amount of explained variance varies considerably between single land covers and even among plots of the same habitat type, as seen for example from wet-season savanna sampling. We assume that this behavior cannot entirely be resolved based on the data presented herein, and therefore, future studies on similar topics might provide valuable insights.

Despite indicated otherwise (Fig. 2), we believe that *p* plays only a minor part in controlling ET. This is not only evidenced by plot-based linear regression (Table 3) but also by the fact that the RF procedure indicates a greater significance almost exclusively for wet-season savanna. Here, *p* typically peaks at around lunchtime and drops to a minimum in the late afternoon (data not shown), thus strongly resembling the diurnal cycle of ET related primarily to *R*_net_. In contrast to the remaining habitats, however, no explicit maximums or minimums are observable during the night (Hardy et al. 1998), thus agreeing with nocturnal ET rates approaching zero.

#### Possible long-term influences

In addition to short-term controls, we assume the discovered ET gradient to be influenced by long-term climatological factors. Associated with the climate gradient are changes in the amount of available moisture, with a gradual increase in annual rainfall from savanna to the grassland level of roughly + 1300 mm (Appelhans et al. 2016). Of course, these spatial differences in moisture availability throughout the year cannot be adequately captured during only a few days of measurement. However, distinct quantitative deviations between dry- and wet-season ET amounts in savanna (Fig. 3e) suggest that water might indeed be a limiting factor at the colline level. This is supported by the results obtained for maize which is traditionally cultivated during the long rains (March to May; Kimaro et al. (2009)) and likewise outweighs ET rates in dry-season savanna by far. As demonstrated by Appelhans et al. (2016, and Fig. 5 therein), the semi-arid savanna receives most of its annual precipitation during the long rains (i.e., wet-season sampling), whereas hardly any rain occurs during the long dry season from June to September (i.e., dry-season sampling). The resulting downregulation of plant stomatal conductance as an adaption of plants to rather dry environments (van den Bergh et al. 2013) might contribute further to attenuated transpiration rates. On the other hand, intra-annual water availability might not be such a crucial factor starting from the submontane level upwards.

Similar limitations have been reported in quite a variety of eco-climatological studies. Biudes et al. (2015), for instance, found that energy exchange processes in Brazil savannas were markedly reduced during the dry season. Moreover, Goulden et al. (2012) reported a rapid ET decline from Sierra Nevada’s montane forest level towards the low-lying savanna areas resulting from less annual rainfall observable even during the wet season.

In high elevations, by contrast, we assume that moisture is not a limiting factor since annual precipitation amounts do not significantly differ from the submontane grassland level (Appelhans et al. 2016). Instead, we expect (i) less *R*_net_ resulting from higher cloud/fog frequencies (Hemp 2009) and topographic exposure (Nyman et al. 2014) and (ii) reduced VPD resulting from lower *T*_a_ to have a dampening effect on ET (van den Bergh et al. 2013). The influence of exposure becomes particularly evident from the west-exposed and rather steep fed1 (Table 1) that receives less incident radiation than the south-exposed fer0 or the flat hel1 plateau.

### Vegetation controls on ET

In terms of vegetation properties, we identify a rather good linear correspondence between ET and NDVI (Fig. 4) documenting that ET in the Kilimanjaro region is not subject to meteorological influences alone. Instead, NDVI-based land cover characteristics seemingly play an important additional role as they explain a considerable amount of variation in mean daily ET rates. Accordingly, the highest NDVI values at the submontane grassland/coffee level and the moderate (massive) decline towards lower (higher) situated plots resemble the vaguely hump-shaped elevation gradient of ET (Fig. 3e). This is not least evidenced by the fact that, when adding NDVI to the already well-performing meteorological model, an additional boost in explained ET variance (Δ*R* ^2^ = + 0.09) can be achieved. In this context, Nagler et al. (2007) already demonstrated that land cover-specific ET could reasonably be estimated as a function of green vegetation derived from EVI (*r* = 0.80 to 0.94) without deploying meteorological drivers. Moreover, the regional validity of our findings is confirmed by Maeda and Hurskainen (2014) who demonstrated that land cover properties as seen from the NDVI could explain between 26 to 39% of additional spatial variation in daytime LST in the Kilimanjaro region. As evidenced from the nearby Taita Hills, Kenya (Maeda et al. 2011), LST exerts a substantial impact on potential and actual ET, thus confirming that the hydrological cycle is subject to a complex interplay of meteorological and land cover-specific factors.

## Conclusions

In the study presented herein, we aimed at establishing a short-term ET-elevation gradient along the highly fragmented southern slopes of Kilimanjaro. Within this scope, the major environmental driving factors of ET should be identified to make a step towards assessing the consequences of local land-use and global climate change on ecosystem functioning through modifications in the regional-scale water budget. Needless to say, such ambitious goals—particularly their generalizability—are hard to accomplish with only a handful of measurement days available. However, we demonstrate that considerable information on the complex interplay between ET and its underlying driving forces may be deduced even from such explicitly short-term observations of meteorological and vegetation-related parameters.

As regards the overall elevation profile, ET revealed a roughly hump-shaped progression curve which, in the short term, was strongly linked to the net radiation budget at each elevation level. Topographic influences and high cloud/fog frequencies attenuated ET at higher elevations, whereas moisture limitations were suspected to exert a restraining effect on water release rates at the low-lying savanna woodlands. Consequently, the highest ET amounts occurred at the submontane coffee/grassland level where neither moisture nor energy limitations could be identified.

In terms of environmental drivers, we found that plot-specific ET amounts could be approximated reasonably well by multivariate regression involving net radiation, soil heat flux, and to a lesser degree, vapor pressure deficit, air pressure (as a surrogate for elevation), and rainfall (*R* ^2^ 0.57 to 1.00). Further gains could be achieved when including vegetation characteristics, resulting in additionally explained variance when comparing pooled data multivariate regression without (*R* ^2^ = 0.71 and 0.78 for hourly and mean daily values, respectively) and with NDVI included (*R* ^2^ = 0.87).

Having identified the main drivers of ET in the area, future work will presumably aim at testing physically based ET modeling approaches to assess the impacts of land-use and climate change on a region-wide scale and over longer time periods. In this context, Camberlin et al. (2014), for instance, demonstrated that seasonal ET_0_ variability at Mt. Kenya strongly depended on seasonal fluctuations of moisture availability, air temperature, and global radiation. Considering the explicitly frequent cloud obscuration and the lack of a long-term ground observation network, setting up a solely remote sensing-based model on an appropriate temporal scale remains a challenging yet not insolvable future goal.

## References

[CR1] Afifi T, Liwenga E, Kwezi L (2014). Rainfall-induced crop failure, food insecurity and out-migration in Same-Kilimanjaro, Tanzania. Climate and Development.

[CR2] Allen, R.G., Pereira, L.S., Raes, D., & Smith, M. (1998). Crop evapotranspiration—guidelines for computing crop water requirements. Irrigation and drainage paper 56, Food and Agriculture Organization of the United Nations, Rome.

[CR3] Altmann A, Toloşi L, Sander O, Lengauer T (2010). Permutation importance: a corrected feature importance measure. Bioinformatics.

[CR4] Appelhans T, Mwangomo E, Otte I, Detsch F, Nauss T, Hemp A (2016). Eco-meteorological characteristics of the southern slopes of Kilimanjaro, Tanzania. International Journal of Climatology.

[CR5] Atzberger C, Eilers PHC (2011). Evaluating the effectiveness of smoothing algorithms in the absence of ground reference measurements. International Journal of Remote Sensing.

[CR6] Biudes MS, Vourlitis GL, Machado NG, de Arruda PHZ, Neves GAR, Lobo FDA, Neale CMU, Nogueira JDS (2015). Patterns of energy exchange for tropical ecosystems across a climate gradient in Mato Grosso, Brazil. Agricultural and Forest Meteorology.

[CR7] Breiman L (2001). Random forests. Machine Learning.

[CR8] Buytaert W, Cuesta-Camacho F, Tobón C (2011). Potential impacts of climate change on the environmental services of humid tropical alpine regions. Global Ecology and Biogeography.

[CR9] Camberlin P, Boyard-Micheau J, Philippon N, Baron C, Leclerc C, Mwongera C (2014). Climatic gradients along the windward slopes of Mount Kenya and their implication for crop risks. Part 1: climate variability. International Journal of Climatology.

[CR10] Cook BI, Smerdon JE, Seager R, Coats S (2014). Global warming and 21st century drying. Climate Dynamics.

[CR11] Da Rocha HR, Goulden ML, Miller SD, Menton MC, Pinto LDVO, De Freitas HC, Silva Figueira AME (2004). Seasonality of water and heat fluxes over a tropical forest in eastern Amazonia. Ecological Applications.

[CR12] Dawson TE (1998). Fog in the California redwood forest: ecosystem inputs and use by plants. Oecologia.

[CR13] DeFries R, Eshleman KN (2004). Land-use change and hydrologic processes: a major focus for the future. Hydrological Processes.

[CR14] Detsch F, Otte I, Appelhans T, Hemp A, Nauss T (2016). Seasonal and long-term vegetation dynamics from 1-km GIMMS-based NDVI time series at Mt. Kilimanjaro, Tanzania. Remote Sensing of Environment.

[CR15] Duane WJ, Pepin NC, Losleben ML, Hardy DR (2008). General characteristics of temperature and humidity variability on Kilimanjaro, Tanzania. Arctic, Antarctic, and Alpine Research.

[CR16] Ensslin A, Rutten G, Pommer U, Zimmermann R, Hemp A, Fischer M (2015). Effects of elevation and land use on the biomass of trees, shrubs and herbs at Mount Kilimanjaro. Ecosphere.

[CR17] Foken T (2008). The energy balance closure problem: an overview. Ecological Applications.

[CR18] Foley JA, DeFries R, Asner GP, Barford C, Bonan G, Carpenter SR, Chapin FS, Coe MT, Daily GC, Gibbs HK, Helkowski JH, Holloway T, Howard EA, Kucharik CJ, Monfreda C, Patz JA, Prentice IC, Ramankutty N, Snyder PK (2005). Global consequences of land use. Science.

[CR19] Glenn EP, Nagler PL, Huete AR (2010). Vegetation index methods for estimating evapotranspiration by remote sensing. Surveys in Geophysics.

[CR20] Google and TerraMetrics (2017). Map data. http://maps.googleapis.com/maps/api/staticmap?center=-3.123553240247,37.366348380164&zoom=10&size=640x497&maptype=satellite&format=gif&sensor=false&scale=2. Accessed 25 July 2017.

[CR21] Goulden ML, Anderson RG, Bales RC, Kelly AE, Meadows M, Winston GC (2012). Evapotranspiration along an elevation gradient in California’s Sierra Nevada. Journal of Geophysical Research: Biogeosciences.

[CR22] Hardwick SR, Toumi R, Pfeifer M, Turner EC, Nilus R, Ewers RM (2015). The relationship between leaf area index and microclimate in tropical forest and oil palm plantation: forest disturbance drives changes in microclimate. Agricultural and Forest Meteorology.

[CR23] Hardy DR, Vuille M, Braun C, Keimig F, Bradley RS (1998). Annual and daily meteorological cycles at high altitude on a tropical mountain. Bulletin of the American Meteorological Society.

[CR24] Hemp A (2005). Climate change-driven forest fires marginalize the impact of ice cap wasting on Kilimanjaro. Global Change Biology.

[CR25] Hemp A (2006). Continuum or zonation? Altitudinal gradients in the forest vegetation of Mt. Kilimanjaro. Plant Ecology.

[CR26] Hemp, A. (2006b). The banana forests of Kilimanjaro: biodiversity and conservation of the Chagga homegardens. Forest diversity and management, D. L. Hawksworth and A. T. Bull, eds., Springer, Dordrecht, The Netherlands, 133–155.

[CR27] Hemp A (2006). Vegetation of Kilimanjaro: hidden endemics and missing bamboo. African Journal of Ecology.

[CR28] Hemp A (2009). Climate change and its impact on the forests of Kilimanjaro. African Journal of Ecology.

[CR29] Jung M, Reichstein M, Ciais P, Seneviratne SI, Sheffield J, Goulden ML, Bonan G, Cescatti A, Chen J, de Jeu R, Dolman AJ, Eugster W, Gerten D, Gianelle D, Gobron N, Heinke J, Kimball J, Law BE, Montagnani L, Mu Q, Mueller B, Oleson K, Papale D, Richardson AD, Roupsard O, Running S, Tomelleri E, Viovy N, Weber U, Williams C, Wood E, Zaehle S, Zhang K (2010). Recent decline in the global land evapotranspiration trend due to limited moisture supply. Nature.

[CR30] Kakane VCK (2004). Soil heat flux-net radiation relations for some surfaces. West African Journal of Applied Ecology.

[CR31] Kimaro AA, Timmer VR, Chamshama SAO, Ngaga YN, Kimaro DA (2009). Competition between maize and pigeonpea in semi-arid Tanzania: effect on yields and nutrition of crops. Agriculture, Ecosystems & Environment.

[CR32] Kite GW, Droogers P (2000). Comparing evapotranspiration estimates from satellites, hydrological models and field data. Journal of Hydrology.

[CR33] Liaw A, Wiener M (2002). Classification and regression by randomForest. R News.

[CR34] Maeda EE, Hurskainen P (2014). Spatiotemporal characterization of land surface temperature in Mount Kilimanjaro using satellite data. Theoretical and Applied Climatology.

[CR35] Maeda EE, Wiberg DA, Pellikka PKE (2011). Estimating reference evapotranspiration using remote sensing and empirical models in a region with limited ground data availability in Kenya. Applied Geography.

[CR36] Meijninger WML, de Bruin HAR (2000). The sensible heat fluxes over irrigated areas in western Turkey determined with a large aperture scintillometer. Journal of Hydrology.

[CR37] Misana SB, Sokoni C, Mbonile MJ (2012). Land-use/cover changes and their drivers on the slopes of Mount Kilimanjaro, Tanzania. Journal of Geography and Regional Planning.

[CR38] Nagler PL, Glenn EP, Kim H, Emmerich W, Scott RL, Huxman TE, Huete AR (2007). Relationship between evapotranspiration and precipitation pulses in a semiarid rangeland estimated by moisture flux towers and MODIS vegetation indices. Journal of Arid Environments.

[CR39] Nakaya K, Suzuki C, Kobayashi T, Ikeda H, Yasuike S (2007). Spatial averaging effect on local flux measurement using a displaced-beam small aperture scintillometer above the forest canopy. Agricultural and Forest Meteorology.

[CR40] Negrón Juárez RI, Hodnett MG, Fu R, Goulden ML, von Randow C (2007). Control of dry season evapotranspiration over the Amazonian forest as inferred from observations at a southern Amazon forest site. Journal of Climate.

[CR41] Nullet D, Juvik JO (1994). Generalised mountain evaporation profiles for tropical and subtropical latitudes. Singapore Journal of Tropical Geography.

[CR42] Nyman P, Sherwin CB, Langhans C, Lane PNJ, Sheridan GJ (2014). Downscaling regional climate data to calculate the radiative index of dryness in complex terrain. Australian Meteorological and Oceanographic Journal.

[CR43] Odhiambo, G.O., & Ain, A. (2011). Comparison of surface layer scintillometer and Eddy covariance footprint and sensible heat flux estimates for different wind directions. In IPCBEE (Ed.) *2nd International Conference on Environmental Science and Technology*, (Vol. 6 pp. V1355–V1360). Singapore: IACSIT Press.

[CR44] Odhiambo GO, Savage MJ (2009). Surface layer scintillometry for estimating the sensible heat flux component of the surface energy balance. South African Journal of Science.

[CR45] Oettli P, Camberlin P (2005). Influence of topography on monthly rainfall distribution over East Africa. Climate Research.

[CR46] Peters MK, Hemp A, Appelhans T, Behler C, Classen A, Detsch F, Ensslin A, Ferger SW, Frederiksen SB, Gebert F, Haas M, Helbig-Bonitz M, Hemp C, Kindeketa WJ, Mwangomo E, Ngereza C, Otte I, Röder J, Rutten G, Costa DS, Tardanico J, Zancolli G, Deckert J, Eardley CD, Peters RS, Rödel M-O, Schleuning M, Ssymank A, Kakengi V, Zhang J, Böhning-Gaese K, Brandl R, Kalko EK, Kleyer M, Nauss T, Tschapka M, Fischer M, Steffan-Dewenter I (2016). Predictors of elevational biodiversity gradients change from single taxa to the multi-taxa community level. Nature Communications.

[CR47] Röder J, Detsch F, Otte I, Appelhans T, Nauss T, Peters MK, Brandl R (2016). Heterogeneous patterns of abundance of epigeic arthropod taxa along a major elevation gradient. Biotropica.

[CR48] Savage MJ (2009). Estimation of evaporation using a dual-beam surface layer scintillometer and component energy balance measurements. Agricultural and Forest Meteorology.

[CR49] Scintec (2013a). Scintec surface layer scintillometer hardware manual real-time extension. Scintec AG, Wilhelm-Maybach-Str. 14, 72108 Rottenburg, Germany, version 1.06.

[CR50] Scintec (2013b). Scintec surface layer scintillometer software manual SRun SLS20/SLS40 SLS20-A/SLS40-A BLS450/BLS900/BLS2000 including SRun real-time sensor interface. Scintec AG, Wilhelm-Maybach-Str. 14, 72108 Rottenburg, Germany, version 1.12.

[CR51] Scintec (2013c). Surface layer scintillometer hardware manual SLS20/ SLS40 SLS20-A/SLS40-A (including SLSDMI option). Scintec AG, Wilhelm-Maybach-Str. 14, 72108 Rottenburg, Germany, version 1.05.

[CR52] Tagesson T, Fensholt R, Guiro I, Rasmussen MO, Huber S, Mbow C, Garcia M, Horion S, Sandholt I, Holm-Rasmussen B, Göttsche FM, Ridler M-E, Olén N, Olsen JL, Ehammer A, Madsen M, Olesen FS, Ardö J (2015). Ecosystem properties of semiarid savanna grassland in West Africa and its relationship with environmental variability. Global Change Biology.

[CR53] Thiermann V, Grassl H (1992). The measurement of turbulent surface-layer fluxes by use of bichromatic scintillation. Boundary-Layer Meteorology.

[CR54] Thompson LG, Mosley-Thompson E, Davis ME, Henderson KA, Brecher HH, Zagorodnov VS, Mashiotta TA, Lin P-N, Mikhalenko VN, Hardy DR, Beer J (2002). Kilimanjaro ice core records: evidence of holocene climate change in tropical Africa. Science.

[CR55] Torbick N, Ge J, Qi J (2009). Changing surface conditions at Kilimanjaro indicated from multiscale imagery. Mountain Research and Development.

[CR56] Tracewski Ł, Butchart SHM, Donald PF, Evans M, Fishpool LDC, Buchanan GM (2016). Patterns of twenty-first century forest loss across a global network of important sites for biodiversity. Remote Sensing in Ecology and Conservation.

[CR57] Tucker CJ (1979). Red and photographic infrared linear combinations for monitoring vegetation. Remote Sensing of Environment.

[CR58] van den Bergh T, Inauen N, Hiltbrunner E, Körner C (2013). Climate and plant cover co-determine the elevational reduction in evapotranspiration in the Swiss Alps. Journal of Hydrology.

[CR59] Van Kesteren B, Beyrich F, Hartogensis OK, van den Kroonenberg AC (2014). The effect of a new calibration procedure on the measurement accuracy of Scintec’s displaced-beam laser scintillometer. Boundary-Layer Meteorology.

[CR60] Weiss A (2002). Determination of thermal stratification and turbulence of the atmospheric surface layer over various types of terrain by optical scintillometry.

